# CT-Based Radiomic Models in Biopsy-Proven Liver Fibrosis Staging: Direct Comparison of Segmentation Types and Organ Inclusion

**DOI:** 10.3390/diagnostics15212671

**Published:** 2025-10-23

**Authors:** Andreea Mihaela Morariu-Barb, Tudor Drugan, Mihai Adrian Socaciu, Horia Stefanescu, Andrei Demirel Morariu, Monica Lupsor-Platon

**Affiliations:** 1 Department of Medical Imaging, “Iuliu Hatieganu” University of Medicine and Pharmacy, 400012 Cluj-Napoca, Romania; andreeambarb@gmail.com (A.M.M.-B.); mihai.socaciu@umfcluj.ro (M.A.S.); monica.lupsor@umfcluj.ro (M.L.-P.); 2 Department of Medical Imaging, Regional Institute of Gastroenterology and Hepatology “Octavian Fodor”, 400162 Cluj-Napoca, Romania; 3 Department of Medical Informatics and Biostatistics, Faculty of Medicine, Iuliu Hațieganu University of Medicine and Pharmacy, 400349 Cluj-Napoca, Romania; 4 Liver Unit, Regional Institute of Gastroenterology and Hepatology “Octavian Fodor”, 400162 Cluj-Napoca, Romania; horia.stefanescu@irgh.ro; 5 Department of Medical Imaging, Emergency County Hospital, 400006 Cluj-Napoca, Romania; morariuandreidemirel@gmail.com

**Keywords:** radiomics, CT scan, 2D segmentation, 3D segmentation, liver and spleen radiomic models, liver fibrosis, liver steatosis

## Abstract

**Background and Objectives**: Liver fibrosis is the key prognostic factor in patients with chronic liver diseases (CLD). Computed tomography (CT) is widely used in clinical practice, but it has limited value in assessing liver fibrosis in precirrhotic stages. Quantitative CT analysis based on radiomics can provide additional information by extracting hidden image patterns, but the optimal approach remains to be determined. The aims of this study were to evaluate automated CT-based radiomic models for predicting biopsy-proven liver fibrosis, to compare different segmentation strategies and organ inclusions approaches, and to assess its performance against vibration-controlled transient elastography (VCTE). We also examined whether these models could predict liver steatosis. **Methods**: In this retrospective study, 58 patients with biopsy-proven CLD and 9 controls underwent VCTE and contrast-enhanced abdominal CT within three months of biopsy. Radiomic features were extracted from portal-venous-phase images using both two-dimensional (2D) and three-dimensional (3D) segmentations of the liver, spleen, and combined liver–spleen. Multilayer perceptron neural (MLP) networks were trained to predict fibrosis staging (≥F1, ≥F2, ≥F3, and F4) and steatosis grading (≥S1, ≥S2, and S3). Model performance was assessed using area under the receiver operating characteristic curve (AUROC) and accuracy. **Results**: The 3D radiomic models outperformed 2D models in predicting liver fibrosis stages. In the 3D radiomic model category, the combined 3D liver–spleen model achieved very good to excellent performance (AUROCs 0.974, 0.929, 0.928, and 0.898, respectively, for ≥F1, ≥F2, ≥F3, and F4), with comparable results to VCTE (AUROCs 0.921, 0.957, 0.968, and 0.909, respectively, for ≥F1, ≥F2, ≥F3, and F4). Radiomic models showed poor predictive ability for steatosis grades (AUROCs 0.44–0.69) compared to controlled attenuation parameter (CAP) (AUROCs 0.798–0.917). **Conclusions**: CT-based radiomic models showed potential for predicting liver fibrosis stage. The 3D model of liver and spleen had the highest performance, comparable to VCTE. This approach could be valuable in clinical settings where elastography is unavailable or inconclusive and for opportunistic screening in patients already undergoing CT for other medical indications. In contrast, portal-venous-phase radiomics lacked predictive value for steatosis assessment. Larger, multicenter studies are required to validate these results.

## 1. Introduction

The global burden of chronic liver disease (CLD) has risen to an estimated 1.5 billion cases [1,2]. Fibrosis is a hallmark of CLD progression and holds strong prognostic value [3]. Fibrosis stage, unlike other histologic features, is the only independent predictor of liver-related events, liver mortality, and overall survival. Compared to patients without fibrosis, those with stage 3 fibrosis had a 14.2-fold higher risk of complications, rising to 51.5-fold in stage 4 [4]. In addition, patients with CLD and advanced fibrosis (F3), even without cirrhosis, have a higher risk of hepatocellular carcinoma than the general population [5].

Although liver biopsy remains the gold standard in assessing the fibrosis stage, its invasiveness and complication risks limit routine or repeated use [6,7]. These limitations have led to the development of non-invasive diagnostic alternatives, including imaging methods such as vibration-controlled transient elastography (VCTE), point or two-dimensional shear-wave elastography (p-SWE and 2D-SWE), and magnetic resonance elastography (MRE) [8,9]. While these tools have demonstrated good diagnostic accuracy, each carries important limitations: MRE is costly, time-consuming, and not widely available, while VCTE and p-SWE/2D-SWE are operator-dependent and may be limited by obesity and ascites [10].

Computed tomography (CT) is one of the most available and widely used imaging modalities globally [11]. Compared to magnetic resonance imaging (MRI), CT is less costly and more accessible and, unlike ultrasound-based techniques, it is not generally limited by obesity or ascites. However, its role in liver fibrosis staging has been primarily to detect morphological changes of cirrhosis, including surface nodularity and hypertrophy of the caudate or left liver lobes [12]. Because many patients with CLD remain asymptomatic until complications occur, individuals with advanced fibrosis (F3–F4), already at increased risk for hepatocellular carcinoma and other adverse outcomes, may go unrecognized on routine CT scans. Identifying patients in precirrhotic stages is critical, as it enables early preventive measures and surveillance strategies to reduce progression and the risk of life-threatening complications. Given its ubiquity, CT holds unique potential for opportunistic screening; patients undergoing CT for unrelated clinical reasons could simultaneously be evaluated for fibrosis, facilitating earlier risk stratification and intervention [13].

Recent advances in quantitative imaging and radiomics have created new opportunities for CT-based assessment of diffuse liver disease [14]. A recent meta-analysis from Wang X et al. has reported good diagnostic accuracy for radiomics in detecting liver fibrosis with AUROCs > 0.90 in the training cohort and >0.89 in validation cohorts, but only 3 studies out of 15 studies included were based on CT imaging [15].

CT-based radiomics for liver fibrosis staging has been explored less extensively than MRI-based radiomics and its optimal methodological approach remains undefined. Prior studies have already addressed some methodological considerations, such as the superior diagnostic performance of contrast-enhanced compared with non-contrast CT and the effect of slice thickness on feature reproducibility [16,17,18]. Nevertheless, important aspects remain to be clarified. Further studies are needed to confirm whether adding splenic features to liver-based models provides additional diagnostic value and to determine whether 2D or 3D segmentation offers the most reliable approach for fibrosis staging [19,20].

This pilot exploratory study is based on the hypothesis that CT radiomics may extend the role of CT beyond morphological liver evaluation, providing a quantitative biomarker for liver fibrosis staging and potentially enabling opportunistic screening in the future. In addition, it seeks to address methodological aspects, including the optimal region of interest and segmentation strategy, to identify the most effective approach for fibrosis assessment.

Therefore, this study aimed to develop and evaluate automated CT-based radiomic models for staging biopsy-proven liver fibrosis. Specifically, we compared different segmentation strategies (2D vs. 3D) and regions of interest (liver, spleen, or both) to identify the most effective approach. We further benchmarked the performance of radiomic models against liver stiffness measurements (LSM) obtained by VCTE in predicting liver fibrosis staging and, also, we assessed their ability to predict biopsy-proven liver steatosis.

## 2. Materials and Methods

### 2.1. Study Population

In this retrospective study, we included 58 patients diagnosed with diffused chronic liver disease based on clinical, laboratory, imaging, and histological criteria. The patients underwent liver biopsy, vibration-controlled transient elastography (VCTE), and a contrast-enhanced abdominal CT scan, all performed within a maximum three-month interval.

Patients with focal liver lesions, especially of large volume and located in the right liver lobe, were excluded from this study, as such findings could potentially interfere with radiomic analysis.

As a control group, we included nine individuals who underwent CT for other medical reasons unrelated to chronic liver disease. These individuals did not undergo biopsy but had normal liver stiffness (no fibrosis) confirmed by VCTE and no clinical, laboratory, or imaging evidence of liver disease.

### 2.2. Histopathological Evaluation

Liver biopsy was performed after obtaining patient consent and evaluating coagulation status. With ultrasound guidance and local anesthesia, a percutaneous approach was used, usually accessing the right liver lobe through the 9th–10th intercostal space.

Histological staging of liver fibrosis was based on the METAVIR score, which classifies fibrosis into five stages: F0 (no fibrosis), F1 (portal fibrosis), F2 (fibrosis with few septa), F3 (bridging fibrosis with numerous septa), and F4 (cirrhosis). Four categories of liver fibrosis were established to standardize the fibrosis severity: early-stage fibrosis (F ≥ 1), significant fibrosis (F ≥ 2), advanced fibrosis (F ≥ 3), and cirrhosis (F4).

Liver steatosis grading was conducted by using the percentage of hepatocytes containing fat droplets: S0 (<5%), S1 (5–33%), S2 (34–66%), and S3 (>66%). Three categories of liver steatosis were established to standardize the severity for model training: at least mild steatosis (S ≥ 1), at least moderate steatosis group (S ≥ 2), and severe steatosis group (S3).

### 2.3. Vibration-Controlled Transient Elastography (VCTE) Evaluation

VCTE examinations were conducted after a fasting period of at least four hours. During a VCTE examination, patients stayed in a supine position with the right arm elevated. The probe was positioned perpendicularly to the patient’s skin, between the 9th and 11th right intercostal space. The result was reliable only when 10 valid readings and an IQR ≤ 30% of the median (IQR/M ≤ 30%) were obtained.

Liver stiffness (LS) cut-offs used to non-invasively stage liver fibrosis were the following: <5.3 for F0, 5.3–7.4 kPa for F1, 7.4–9.1 kPa for F2, 9.1–13.2 kPa for F3, and ≥13.2 kPa for cirrhosis (F4) [21].

Liver steatosis was also assessed non-invasively by measuring the controlled attenuation parameter (CAP) during VCTE examination. CAP cut-offs applied to categorize steatosis severity were the following: ≥248 dB/m for mild steatosis (S1), ≥268 dB/m for moderate steatosis (S2), and ≥280 dB/m for severe steatosis (S3) [22].

### 2.4. CT Imaging Protocol

All patients underwent multiphase contrast-enhanced abdominal CT imaging using a Somatom Perspective 64-slice CT scanner (Siemens Healthineers, Erlangen, Germany; software version syngo CT VA48A, 2015 model). The protocol included non-contrast, arterial, and portal-venous phases, with each phase acquired at a slice thickness of 3 mm. Following anonymization, Digital Imaging and Communications in Medicine (DICOM) files were retrieved from the Picture Archiving and Communication System (PACS) for radiomic analysis.

#### 2.4.1. Image Segmentation and Radiomic Feature Extraction

Image segmentation was performed using the open-source Slicer software (version 5.6.2) in the portal-venous phase. All radiomic preprocessing and feature extraction were performed using the Radiomics extension, which implements the PyRadiomics (version 3.1.0) library compliant with the Image Biomarker Standardisation Initiative (IBSI) definitions. Accordingly, CT images and corresponding segmentation masks were resampled to isotropic voxel spacing of 1 × 1 × 1 mm^3^ using the default interpolation methods implemented in the software (B-spline interpolation for images and nearest-neighbor interpolation for masks). Image intensities were discretized using a fixed bin width of 25 Hounsfield units (HU) prior to texture feature computation. No additional intensity normalization was applied, as all scans were acquired using standardized CT protocols and voxel values expressed in Hounsfield units are inherently comparable across patients and scanners. No additional filters (such as wavelet or Laplacian of Gaussian) were applied.

**Figure 1:**
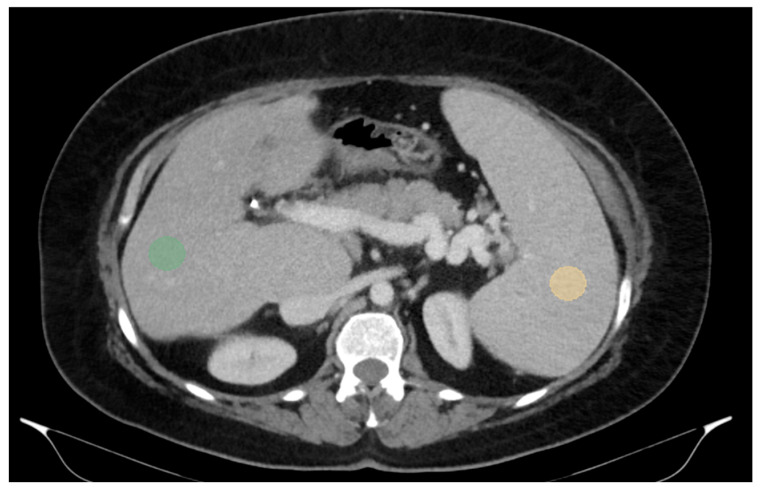
Abdominal portal-venous-phase CT scan, axial plane. Example of 2D liver and spleen segmentations. Two-dimensional liver and spleen segmentations were obtained by placing regions of interest (ROIs) in the right liver lobe at the level of the 9th right intercostal space (green ROI) and in the spleen (orange ROI), while avoiding major vascular structures.

For two-dimensional (2D) segmentation, regions of interest (ROIs) were manually delineated on the portal-venous-phase images. In the liver, ROIs were placed in the right lobe, at the level of 9th to 10th intercostal space, approximately 10 mm below the liver capsule and avoiding large vessels, which is also the typical site for liver biopsy and VCTE. Spleen ROIs were similarly delineated while avoiding major vascular structures (Figure 1).

**Figure 2:**
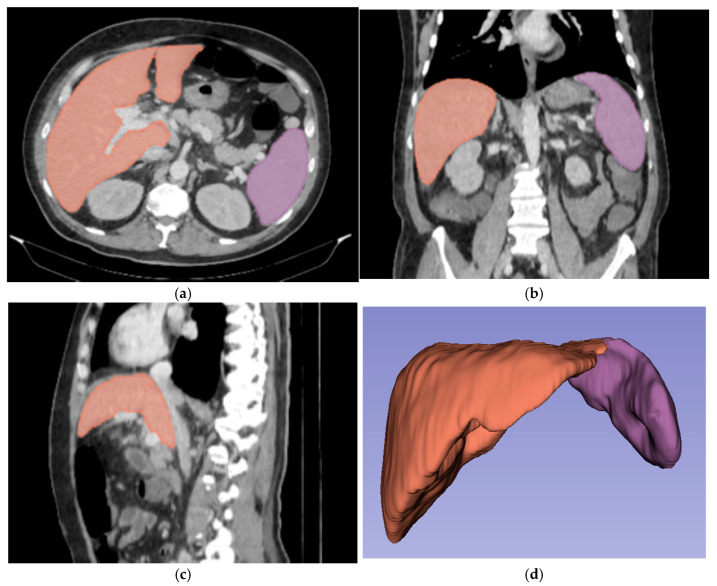
Abdominal portal-venous-phase CT scan: axial, coronal, and sagittal planes (**a**–**c**). Example of 3D liver and spleen segmentations. Three-dimensional liver and spleen segmentations were performed by using the TotalSegmentator extension in Slicer software, followed by manual corrections. Images (**a**–**c**) show the liver and spleen segmentation in axial, coronal, and sagittal planes. Image (**d**) shows the final 3D segmentation of these organs.

For three-dimensional (3D) segmentation, the TotalSegmentator module in Slicer was used for automated organ segmentation, followed by manual correction of the right and left portal vein, to ensure anatomical accuracy (Figure 2).

Radiomic features were extracted using the PyRadiomics extension (v3.1.0.) integrated within Slicer software, with bin width of 25.00. Features were grouped according to the segmented organ (liver or spleen) and the segmentation type (2D or 3D). Each group included approximately 107 features, which were categorized into several families: first-order statistics, shape features, gray-level co-occurrence matrix (GLCM), gray-level run length matrix (GLRLM), gray-level size zone matrix (GLSZM), gray-level dependence matrix (GLDM), and neighboring gray tone difference matrix (NGTDM). All of these features constituted the input for the neural networks.

#### 2.4.2. Radiomic Model Development for Liver Fibrosis Prediction

After image segmentation and radiomic feature extraction, multilayer perceptron (MLP) neural networks in SPSS program were trained to classify fibrosis severity. The output of the models was fibrosis stage, categorized into four categories: no fibrosis, significant fibrosis, advanced fibrosis, and cirrhosis. Separate models were developed for each of the six segmentation configurations: 2D liver, 2D spleen, combined 2D liver and spleen, 3D liver, 3D spleen, and combined 3D liver and spleen.

Due to prior splenectomy, radiomic spleen features were unavailable for two patients.

#### 2.4.3. Radiomic Model Development for Liver Steatosis Prediction

In a separate analysis, MLP models were also trained to predict liver steatosis grades: no steatosis vs. any grade of steatosis (S0 vs. S1, 2, and 3), at least moderate steatosis (S0 and 1 vs. S 2 and 3), and severe steatosis (S0, 1, and 2 vs. S3).

### 2.5. Statistical Analysis

The PyRadiomics software provided a large number of features (107 per examination), grouped into seven categories: shape-based features, first-order intensity statistics, gray-level co-occurrence matrix, gray-level dependence matrix, gray-level run length matrix, gray-level size zone matrix, and neighborhood gray tone difference matrix. This high dimensionality posed a major challenge for statistical analysis and classification. The complete list of radiomic features used is provided in the Supplementary Material.

Conventional statistical approaches—logistic regression with LASSO or Ridge regularization, linear/quadratic discriminant analysis, principal component analysis, and partial least squares discriminant analysis—did not yield usable models. Consequently, we employed machine learning, specifically deep neural networks.

Classification was performed using the neural network module of IBM SPSS v25 with a random 70/30 training–testing split. SPSS automatically handled data partitioning for training and validation. The architecture included two hidden layers (20 and 15 neurons, respectively). Input variables were rescaled to a standardized range before training. The optimization employed the scaled conjugate gradient algorithm in batch mode, ensuring efficient convergence. Convergence was monitored through multiple stopping criteria: an error change smaller than 0.0001, an error ratio of 0.001, no improvement for one consecutive step, or a maximum training time of 15 min. Model performance was evaluated through classification accuracy, receiver operating characteristic analysis, and variable importance measures. Cases with user-defined missing values were excluded from the analysis to preserve model consistency and avoid bias. The process was performed 5 times per each sample on different randomly selected batches of data, and the results were averaged to increase accuracy.

Given the small sample size and software constraints, no weighting procedures were applied to correct for data imbalance, which we recognize as a limitation of our pilot study.

### 2.6. Ethical Considerations

Patient data were retrospectively collected from the electronic medical records of the “Octavian Fodor” Regional Institute of Gastroenterology and Hepatology, Cluj-Napoca, Romania. All data were anonymized before analysis, and strict confidentiality was assured. At the time of admission, patients had provided written consent for the use of their medical information in research. Therefore, separate approval from the ethics committee was not required, in accordance with the hospital’s regulations.

## 3. Results

We included 67 patients, 58 diagnosed with chronic liver diseases and 9 controls with normal liver stiffness (F0) confirmed by VCTE and no clinical, laboratory, or imaging evidence of liver disease. The characteristics of the study cohort are shown in Table 1.

### 3.1. Assessment of Liver Fibrosis

#### 3.1.1. Performance of CT-Based Radiomic Models in Prediction of Biopsy-Proven Liver Fibrosis

**Figure 3:**
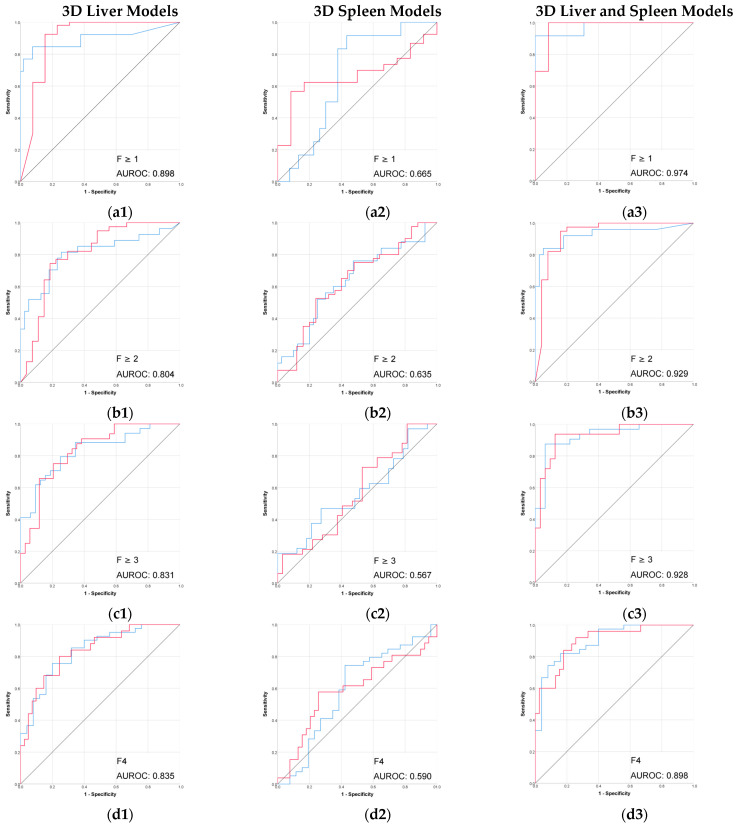
Performance (ROC) of the 3D CT-based radiomic models for prediction of liver fibrosis stages. (**a1**–**a3**) F ≥ 1, (**b1**–**b3**) F ≥ 2, (**c1**–**c3**) F ≥ 3, (**d1**–**d3**) F4. Red line = “Yes” class (presence of fibrosis ≥ stage threshold), blue line = “No” class (no fibrosis or fibrosis < stage threshold). The AUROC is reported for the positive class.

**Figure 4:**
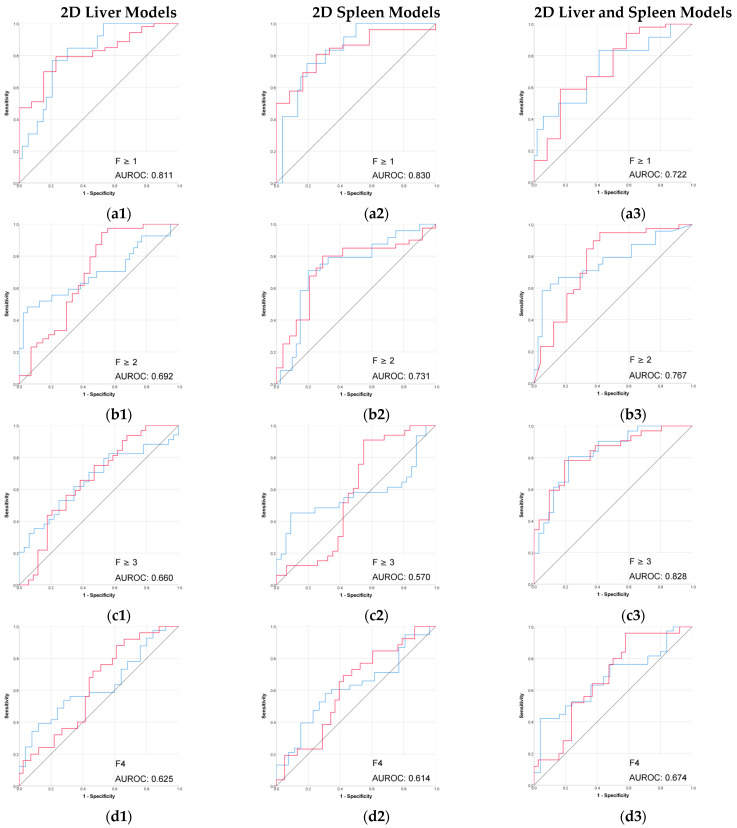
Performance (ROC) of the 2D CT-based radiomic models for prediction of liver fibrosis stages. (**a1**–**a3**) F ≥ 1, (**b1**–**b3**) F ≥ 2, (**c1**–**c3**) F ≥ 3, (**d1**–**d3**) F4. Red line = “Yes” class (presence of fibrosis ≥ stage threshold), blue line = “No” class (no fibrosis or fibrosis < stage threshold). The AUROC is reported for the positive class.

The radiomic models based on 3D segmentation outperformed 2D models in the prediction of liver fibrosis groups (Table 2, Figure 3 and Figure 4).

The combined 3D liver and spleen model was superior to the single-organ models. The highest AUROC and accuracy (0.974 and 95%) of this 3D combined model was observed in discriminating between no fibrosis versus any stage of fibrosis (F ≥ 1), while performance remained strong for advanced fibrosis and cirrhosis. Among the single-organ models, the 3D liver model showed the highest performance, with AUROC values ranging from 0.804 to 0.898 and accuracies up to 92%.

The performance of 2D models in the prediction of liver fibrosis categories was more variable and generally lower than the performance of 3D models. The 2D spleen model achieved a good performance only for F ≥ 1 (AUROC, 0.830; accuracy, 91.3%). Combining 2D liver and spleen features modestly improved performance for the advanced fibrosis (AUROC, 0.828; accuracy, 64.7%) but remained below the performance of the corresponding 3D models.

#### 3.1.2. Performance of VCTE Liver Stiffness Measurements (LSM) in Prediction of Biopsy-Proven Liver Fibrosis

**Figure 5:**
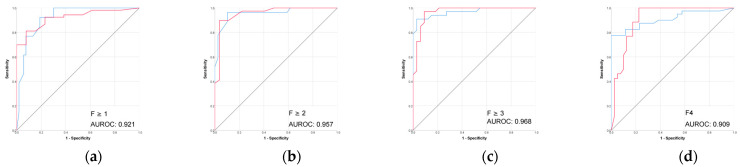
(**a**–**d**) Performance (ROC) of VCTE liver stiffness in prediction of biopsy-proven liver fibrosis. Red line = “Yes” class (presence of fibrosis ≥ stage threshold), blue line = “No” class (no fibrosis or fibrosis < stage threshold). The AUROC is reported for the positive class.

The performance of VCTE was excellent across all fibrosis stages (Table 3 and Figure 5). The highest performance was seen for advanced fibrosis prediction (F ≥ 3) with an AUROC of 0.968 and accuracy of 88.2%.

The combined 3D liver–spleen model performed comparably to VCTE across fibrosis stages, with slightly higher performance in detecting early-stage fibrosis and slightly lower performance in more advanced stages.

### 3.2. Assessment of Liver Steatosis

#### 3.2.1. Performance of CT-Based Radiomic Models in Prediction of Biopsy-Proven Liver Steatosis Grades

All radiomic models showed very low performances in predicting histological steatosis grades, with AUROCs ranging between 0.44 and 0.69, regarding the segmentation types or the segmented organ (Table 4).

#### 3.2.2. Performance of Controlled Attenuated Parameter (VCTE-CAP) in Prediction of Biopsy-Proven Liver Steatosis Grades

**Figure 6:**
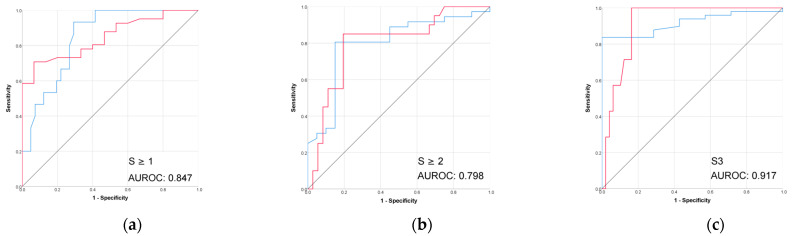
(**a**–**c**) The performance of VCTE-CAP in prediction of biopsy-proven liver steatosis. Red line = “Yes” class (presence of steatosis ≥ stage threshold), blue line = “No” class (no steatosis or steatosis < stage threshold). The AUROC is reported for the positive class.

The performance of controlled attenuated parameter (CAP) in the prediction of biopsy-proven liver steatosis grades was better than all the radiomic models, as shown in Table 5 and Figure 6.

## 4. Discussion

This study aimed to assess the potential of quantitative CT-based radiomic analysis as an alternative non-invasive method for staging liver fibrosis. To better understand how different approaches of imaging segmentation and organ inclusion influence radiomic model performance, we directly compared two segmentation types (3D and 2D) and single-organ (liver or spleen) versus combined liver–spleen models. We considered these comparisons from a practical perspective; 3D segmentation and multi-organ analysis are technically more complex and time-consuming and 2D segmentation and single-organ analysis may be easier to integrate into clinical practice. Our results, however, showed that CT radiomic models can predict liver fibrosis stage, but the best performance is achieved using 3D segmentation of both liver and spleen.

This study also explored the potential of CT-based radiomic analysis for predicting liver steatosis grades, given the frequent coexistence of steatosis and fibrosis in CLD. In our cohort, however, radiomic models of the liver and/or spleen based on the portal-venous-phase CT scan demonstrated poor performance for steatosis grading, underscoring their particular relevance for predicting liver fibrosis.

These findings should be interpreted in the context of a small, single-center pilot cohort, designed primarily to explore feasibility. As such, the results are promising but preliminary, requiring validation in larger, independent populations before broader conclusions can be drawn.

### 4.1. Liver Fibrosis Staging

The performance of conventional CT imaging for staging liver fibrosis is limited, particularly in precirrhotic stages. However, since CT is one of the most widely used imaging methods worldwide [11], it provides a unique opportunity to incidentally detect clinically important liver fibrosis in asymptomatic patients. Many individuals undergo CT scans for other medical reasons than liver disease. Therefore, adding radiomic analysis to routine imaging interpretation could help identify liver fibrosis in people who might otherwise go undiagnosed. This could be particularly important in at-risk populations, where early detection and treatment can change the course of the disease.

In our study, 3D radiomic models outperformed 2D models in predicting liver fibrosis stages. This superiority likely reflects the diffuse and heterogeneous nature of fibrosis, which may be underestimated by a 2D region of interest. In cirrhosis, the liver will go through global morphological changes, such as a nodular surface and a dysmorphic shape, with right lobe atrophy and caudate and left lobe hypertrophy. These changes are more accurately reflected by a 3D segmentation, which can assess the entire organ.

Even so, the 2D liver and 2D spleen models still performed well in differentiating patients without fibrosis from those with any stage of fibrosis (AUROCs, 0.811 and 0.830; accuracies, 80% and 91.3%). This suggests that 2D models could play a role in a stepwise diagnostic approach, as an initial tool to “rule out” fibrosis.

The interplay between the liver and spleen is clinically important in chronic liver disease, as splenomegaly represents an indirect sign of portal hypertension. Consistent with this, the combined 3D liver–spleen model outperformed the one-organ 3D radiomic models.

The 3D spleen model alone showed weak performance in predicting fibrosis stages. Moreover, we included some patients with PSVD, who presented with splenomegaly but only minimal or early fibrosis, which may have influenced the results of this radiomic model. Interestingly, the 2D spleen model performed well for differentiating F0 vs. F ≥ 1, supporting the idea that spleen radiomics may capture subtle, otherwise imperceptible changes that develop in parallel with the liver’s fibrosis progression.

Previous CT-based radiomic studies have reported good performance in staging liver fibrosis, with AUROCs ranging from 0.723 to 1.000, but differ considerably in the CT phase that was used for segmentation, segmentation method, target organ, and feature selection, making direct comparison challenging [16,20]. The present study focused on the predictive value of imaging characteristics, without any influence of clinical or laboratory information. Therefore, all current results were obtained without adding other data into the radiomic models, which may partly explain the differences between these findings and those previously reported in the literature. Our 3D models based on CT portal venous phase showed very good performances, with AUROCs broadly in line with previous CT-based radiomic studies that used this type of segmentation. For the prediction of F ≥ 1, the combined 3D model (AUROC 0.974; accuracy 95%) outperformed the combined liver–spleen model reported by Yin et al. (AUROC 0.92) and was comparable with the results reported by Tang et al., even though their models incorporated clinical data [19,20]. Our results for advanced fibrosis and cirrhosis prediction (AUROCs 0.928 and 0.898, respectively) were also aligned with the high ranges reported in the recent meta-analysis [15].

In this study, the 2D models generally performed weaker than 3D models in predicting liver fibrosis stage. When compared to other similar studies, such as the multi-institutional work of Wang et al. [23], our results were slightly higher but still broadly in line with theirs.

VCTE is the most validated non-invasive method for assessing liver fibrosis stage, with very good performances, a fact also shown in the present study (AUROCs > 0.90 and accuracies > 80% for the prediction of all fibrosis stages) [24]. Compared to VCTE, the CT-based radiomic models, particularly the combined 3D liver and spleen model, showed competitive performance across fibrosis stages but with lower accuracies in the more advanced categories. In current practice, VCTE has some limitations, such as high BMI or the presence of ascites [3]. As the incidence and prevalence of obesity and MASLD have been constantly increasing in recent years and MASLD is expected to become the most common etiology of CLD, these limitations are likely to become even more important in the near future [25]. New methods that can overcome these limitations are needed, especially in this context. Although VCTE remains a strong non-invasive method, this study highlights the potential of CT-based radiomics as a viable alternative, particularly in settings where VCTE is unavailable or technically limited or in patients undergoing CT for other indications, who may thereby obtain additional information on the stage of CLD. Our study did not address the influence of other CT-related factors on radiomic models in this setting, such as unenhanced or arterial CT phases or CT slice thickness (thin versus thick slices). Hu et al. [18] reported that radiomic models based on entire liver segmentation from portal-venous-phase thin-slice CT images (1.3–2 mm) performed better than those from thick slices (5–7 mm) for staging liver fibrosis. We used in this study 3 mm slices. The performances of our 3D liver models were higher than those of radiomic models based on thick slices (AUROCs 0.804, 0.831, and 0.835 vs. 0.781, 0.719, and 0.852) and lower than those obtained from thin slices for significant fibrosis (0.80 vs. 0.901), advanced fibrosis (0.804 vs. 0.851), and cirrhosis (0.835 vs. 0.937).

### 4.2. Liver Steatosis Grading

In chronic liver diseases, fibrosis and steatosis are histological features that frequently coexist. Imaging can be influenced by both, so a method with high specificity for each of these features is needed for accurate characterization of CLD. The VCTE-controlled attenuation parameter (CAP) is a validated tool for assessing liver fat content in clinical practice. A large meta-analysis including patients with mixed CLD etiologies reported good performance, with AUROCs of 0.823, 0.865, and 0.882, respectively, for steatosis grades ≥ S1, ≥S2, and S3 prediction [22].

In our study, CAP also performed well in detecting mild and moderate steatosis (AUROCs of 0.847 and 0.798) and very well for severe steatosis prediction (AUROC 0.917). However, the higher performance in the severe steatosis subgroup may have been influenced by its small sample size.

CT-based radiomic models performed poorly in predicting steatosis grades in the current study. This indirectly suggests that the radiomic features extracted from portal-venous-phase CT images are not influenced by hepatic fat content. This contrasts with recent studies showing CT radiomics can predict steatosis grades, although these studies used unenhanced CT images for segmentation and they also combined radiomic features with clinical data [26,27]. Moreover, it is also well known that conventional liver steatosis assessment on CT scans (based on Hounsfield units) is more accurate on unenhanced scans [28]. Taken together, these observations suggest that unenhanced CT-based radiomics could be more suitable for grading steatosis, while contrast-enhanced CT-based radiomics may be more useful for staging fibrosis. Future studies with larger and more balanced cohorts are needed to clarify whether the poor steatosis performance observed here reflects a true limitation of portal-venous-phase radiomics or simply the constraints of our dataset.

This study has several notable strengths. First, liver fibrosis and steatosis were staged and graded against histology, which remains the gold-standard reference. Second, by comparing different segmentation strategies within the same cohort and showing how these can influence performance, this study provides valuable insight into the optimal imaging approach in radiomic analysis for fibrosis staging. Moreover, model performance was also compared with VCTE, the most validated non-invasive modality for fibrosis staging, which enhances the clinical relevance of our findings. Finally, both fibrosis and steatosis were assessed in the same cohort, offering a more comprehensive evaluation of CLD using radiomics.

Although this is a preliminary study, our findings provide insight into the role of radiomics in liver fibrosis staging, showing that 3D segmentation of both the liver and spleen, despite being more difficult to perform and more time-consuming than 2D or single-organ approaches, offers superior performance. These findings need to be further validated in larger patient cohorts.

This study has several limitations that should be acknowledged. First, its retrospective design carries the risk of selection bias and limits causal interpretation. The sample size was small, especially in some fibrosis stages or steatosis grades, which may affect the generalizability of the radiomic models. The imbalance between groups may reduce the robustness of subgroup analyses and may bias the reported AUROC values, potentially leading to an overestimation of performance in categories with fewer cases.

Although a well-established segmentation protocol was followed, any manual steps in 2D or 3D segmentation could introduce an operator-dependent variability that may affect reproducibility. To minimize this, we performed measurements as objectively as possible by using anatomical landmarks for 2D segmentation of liver and spleen, such as the 9th–10th intercostal space, approximately 10 mm below the liver capsule, while avoiding large vessels, and by removing the right and left portal veins from the 3D segmentation generated with TotalSegmentator. Nevertheless, segmentation remains a critical step in radiomics, and differences in operator experience or software tools could introduce variability across centers.

In addition, although neural networks were appropriate to be used to manage the large volume of data, these methods have certain drawbacks, including their “black box” nature, which limits interpretability and their tendency to overfitting. We attempted to identify the most relevant radiomic variables that contributed to model performance, with the aim of using them in more targeted analyses or classical statistical approaches. However, there was no consistent overlap across models, which prevented us from highlighting specific variables as being particularly influential. This lack of transparency limits the scientific interpretability of the models and represents an inherent challenge of deep learning methods.

This was a single-center study and external validation in independent, multicenter cohorts is needed to confirm our findings. Without such validation, the reproducibility of our results remains uncertain and the generalizability of the models to broader populations cannot be assumed.

## 5. Conclusions

The results of this pilot study highlight the potential of CT-based radiomics as a promising, non-invasive tool for liver fibrosis staging, as well as the value of 3D segmentation and the inclusion of both liver and spleen features in the analysis.

The 3D radiomic models showed better performance than the 2D models in predicting liver fibrosis stages and combining a 3D liver and spleen provided improved results compared with models based on only one organ. The combined 3D liver–spleen model performed comparably to VCTE across fibrosis stages, suggesting it could be useful when elastography is not available or gives inconclusive results. Furthermore, in patients who undergo CT for other indications, applying such an algorithm could provide valuable additional information on the stage of chronic liver disease. In contrast, contrast-enhanced CT-based radiomics did not predict hepatic steatosis, and this supports their likely predilection for fibrosis changes, an important aspect in current practice, since these two conditions often coexist.

These findings should be interpreted in the context of a small, single-center pilot cohort, designed primarily to explore feasibility. As such, the results are promising but preliminary, requiring validation in larger, multicenter, and independent populations to confirm the reproducibility and clinical applicability of these models.

## Figures and Tables

**Table 1:** Characteristics of the study cohort.

Category	Variable	Value (Number, Mean, %)
Demographics	Age	Median 58 years
	Sex	37 males (55%) 30 females (45%)
Liver disease etiology	Viral hepatitis	29%
	Alcohol-related liver disease (ALD)	26%
	Porto-sinusoidal vascular disorder (PSVD)	10%
	Metabolic-associated steatotic liver disease (MASLD)	9%
	Autoimmune hepatitis	5%
Histological fibrosis stages (METAVIR score)	F0	4 (6.9%)
	F1	14 (24.1%)
	F2	7 (12.1%)
	F3	7 (12.1%)
	F4	26 (44.8%)
Histological steatosis grades (% of fat-containing hepatocytes)	S0	17 (32%)
	S1	23 (43.3%)
	S2	10 (18.8%)
	S3	3 (5.6%)
VCTE results	Liver stiffness (LS), kPa	Mean 24.5 (3.0–75.0)
	Controlled attenuation parameter (CAP), dB/m	Mean 246 (126–374)

**Table 2:** Performances of CT-based radiomic models for the prediction of liver fibrosis stages.

Portal-Venous-Phase Segmentation	Organ	F ≥ 1		F ≥ 2		F ≥ 3		F4	
		AUROC	Accuracy	AUROC	Accuracy	AUROC	Accuracy	AUROC	Accuracy
3D radiomic model	Liver	0.898	92%	0.804	60%	0.831	68%	0.835	72%
	Spleen	0.665	82.4%	0.635	52.9%	0.567	58.8%	0.590	58.8%
	Liver and Spleen	0.974	95%	0.929	75%	0.928	75%	0.898	65%
2D radiomic model	Liver	0.811	80%	0.692	60%	0.660	45%	0.625	80%
	Spleen	0.830	91.3%	0.731	60.9%	0.570	69.6%	0.614	52.2%
	Liver and Spleen	0.722	82.4%	0.767	70.6%	0.828	64.7%	0.674	52.9%

**Table 3:** Performance of VCTE liver stiffness in prediction of biopsy-proven liver fibrosis.

Fibrosis Stage	F ≥ 1		F ≥ 2		F ≥ 3		F4	
VCTE-LSM (kPa)	AUROC	Accuracy	AUROC	Accuracy	AUROC	Accuracy	AUROC	Accuracy
	0.921	88.2%	0.957	94.1%	0.968	88.2%	0.909	82.5%

**Table 4:** Performance of CT-based radiomic models in the prediction of biopsy-proven liver steatosis grades.

Portal-Venous-Phase Segmentation	Organ	S ≥ 1		S ≥ 2		S3	
		AUROC	Accuracy	AUROC	Accuracy	AUROC	Accuracy
3D radiomic model	Liver	0.680	70.4%	0.574	66.7%	0.642	88.5%
	Spleen	0.693	71.4%	0.674	66.7%	0.458	79.2%
	Liver and Spleen	0.646	70.6%	0.532	52.9%	0.669	94.4%
2D radiomic model	Liver	0.440	66.7%	0.561	80%	0.613	86.4%
	Spleen	0.695	68.8%	0.658	50%	0.627	85%
	Liver and Spleen	0.632	68.4%	0.607	52.6%	0.630	90.5%

**Table 5:** Performance of controlled attenuated parameter (CAP) in the prediction of biopsy-proven liver steatosis grades.

Steatosis Stage	S ≥ 1		S ≥ 2		S3	
VCTE-CAP (dB/m)	AUROC	Accuracy	AUROC	Accuracy	AUROC	Accuracy
	0.847	83.3%	0.798	77.8%	0.917	72.2%

## Data Availability

The original contributions presented in the study are included in the article material; further inquiries can be directed to the corresponding author.

## Supplementary Materials

The following supporting information can be downloaded at: https://www.mdpi.com/article/10.3390/diagnostics15212671/s1, Document S1: manuscript-supplementary.docx.

## Abbreviations

The following abbreviations are used in this manuscript:

CLD	Chronic liver disease
CT	Computed tomography
2D	Two-dimensional
3D	Three-dimensional
VCTE	Vibration-controlled transient elastography
MLP	Multilayer perceptron
AUROC	Area Under the Receiver Operating Characteristic curve
CAP	Controlled attenuation parameter
p-SWE	Point shear-wave elastography
2D-SWE	Two-dimensional shear-wave elastography
MRE	Magnetic resonance elastography
MRI	Magnetic resonance imaging
LSM	Liver stiffness measurements
ROI	Region of interest
DICOM	Digital Imaging and Communications in Medicine
PACS	Picture Archiving and Communication System
GLCM	Gray-level co-occurrence matrix
GLRLM	Gray-level run length matrix
GLSZM	Gray-level size zone matrix
GLDM	Gray-level dependence matrix
NGTDM	Neighboring gray tone difference matrix
ALD	Alcohol-related liver disease
PSVD	Porto-sinusoidal vascular disease
MASLD	Metabolic-associated steatotic liver disease
