# Supplementary material for: CT-Based Radiomic Models in Biopsy-Proven Liver Fibrosis Staging: Direct Comparison of Segmentation Types and Organ Inclusion
Source: Diagnostics (Basel). 2025 Oct 23;15(21):2671. doi: 10.3390/diagnostics15212671 (PMC12607452; doi:10.3390/diagnostics15212671)
Supplement: Supplementary file 1 [file diagnostics-15-02671-s001.zip › diagnostics-3882670-supplementary.pdf]

The suite of radiomics features used.

1. Shape-based features

These describe the **geometry** of the segmented lesion/ROI (independent of intensity).

- **Elongation** – measures how stretched the shape is (ratio of the second-largest to the largest principal axis length; close to 1 = round, close to 0 = elongated).
- **Flatness** – how flat the object is (ratio of smallest to largest principal axis).
- **LeastAxisLength** – length of the shortest principal axis of the object (from PCA).
- **MinorAxisLength** – length of the second-largest principal axis.
- **MajorAxisLength** – length of the largest principal axis.
- **Maximum2DDiameterColumn** – largest distance between any two voxels in the same **row direction** (y-axis).
- **Maximum2DDiameterRow** – largest distance in the **column direction** (x-axis).
- **Maximum2DDiameterSlice** – largest in-plane distance within a 2D slice.
- **Maximum3DDiameter** – largest Euclidean distance between any two voxels in the whole 3D volume.
- **MeshVolume** – physical volume computed from the triangulated surface mesh of the ROI.
- **SurfaceArea** – total area of the 3D surface boundary.
- **SurfaceVolumeRatio** – compactness measure: SA / Volume. Lower values = more spherical.
- **Sphericity** – how spherical the shape is, based on ratio of volume to surface area compared with a sphere.
- **VoxelVolume** – physical volume of one voxel (depends on voxel spacing).

2. First-order intensity statistics

These summarize **intensity distribution** inside the ROI (like histogram stats).

- **@10Percentile / @90Percentile** – intensity values at 10th and 90th percentile.
- **Energy** – sum of squared intensities (reflects signal magnitude).
- **Entropy** – randomness of intensity distribution.
- **InterquartileRange** – difference between 75th and 25th percentiles.
- **Kurtosis** – "peakedness" of the histogram (high = heavy tails/sharp peak).
- **Maximum / Minimum** – max and min intensity.
- **Mean** – average intensity.

- **Median** – median intensity.
- **MeanAbsoluteDeviation** – mean of absolute differences from the mean.
- **RobustMeanAbsoluteDeviation** – same but using intensities between 10–90th percentile (robust to outliers).
- **Range** – max − min intensity.
- **RootMeanSquared** – square root of mean of squared intensities (measure of magnitude).
- **Skewness** – asymmetry of histogram (positive = right tail longer).
- **TotalEnergy** – Energy × voxel volume (scale-invariant).
- **Uniformity** – sum of squared probabilities of intensity levels (inverse of entropy).
- **Variance** – spread of intensities.

3. Gray Level Co-occurrence Matrix features

These capture **texture patterns** by analyzing how often gray-level pairs occur in neighboring voxels.

- **Autocorrelation** – linear dependency of gray levels.
- **ClusterProminence** – skewness of clusters (higher = more asymmetry).
- **ClusterShade** – asymmetry in gray-level clustering.
- **ClusterTendency** – tendency of pixels to form clusters.
- **Contrast** – local intensity variation.
- **Correlation** – linear correlation of neighboring gray levels.
- **DifferenceAverage / DifferenceEntropy / DifferenceVariance** – features of intensity differences between neighbors.
- **Id, Idm, Idn, Idmn** – "Inverse Difference" measures: homogeneity (higher = smoother texture). Variants are normalized or squared.
- **Imc1, Imc2** – "Informational Measure of Correlation": redundancy vs independence in texture.
- **InverseVariance** – homogeneity emphasizing smaller gray-level differences.
- **JointAverage / JointEnergy / JointEntropy** – stats from the joint probability distribution of gray-level pairs.
- **MCC** – maximal correlation coefficient (complex dependency measure).
- **MaximumProbability** – largest probability in GLCM (dominant texture).
- **SumAverage / SumEntropy / SumSquares** – stats of sums of gray levels.

4. Gray Level Dependence Matrix features

Describe how many neighboring voxels are dependent on a center voxel.

- **DependenceEntropy** – randomness of dependencies.
- **DependenceVariance** – spread of dependence sizes.
- **DependenceNonUniformity / Normalized** – heterogeneity of dependence sizes.
- **GrayLevelNonUniformity** – gray-level distribution non-uniformity.
- **GrayLevelVariance** – variance of gray levels.
- **HighGrayLevelEmphasis** – emphasizes high gray levels.
- **LargeDependenceEmphasis** – emphasizes large homogeneous regions.
- **LargeDependenceHighGrayLevelEmphasis / LowGrayLevelEmphasis** – interaction of large dependencies with high/low gray levels.
- **LowGrayLevelEmphasis** – highlights low gray-level structures.
- **SmallDependenceEmphasis** – emphasizes fine textures.
- **SmallDependenceHighGrayLevelEmphasis / LowGrayLevelEmphasis** – same but weighted by intensity.

5. Gray Level Run Length Matrix features

Describe consecutive runs of voxels with same intensity.

- **GrayLevelNonUniformity / Normalized** – heterogeneity in gray levels.
- **GrayLevelVariance** – variance of gray levels.
- **HighGrayLevelRunEmphasis** – long runs of high gray levels.
- **LongRunEmphasis** – emphasizes long consecutive runs.
- **LongRunHighGrayLevelEmphasis / LowGrayLevelEmphasis** – long runs with high/low intensities.
- **LowGrayLevelRunEmphasis** – emphasizes runs of low intensity.
- **RunEntropy** – randomness of run distribution.
- **RunLengthNonUniformity / Normalized** – heterogeneity of run lengths.
- **RunPercentage** – proportion of short vs long runs.
- **RunVariance** – variance of run lengths.
- **ShortRunEmphasis** – emphasizes short runs (fine textures).
- **ShortRunHighGrayLevelEmphasis / LowGrayLevelEmphasis** – short runs with high/low intensity.

6. Gray Level Size Zone Matrix features

Similar to GLRLM, but zones are connected 2D/3D areas with same gray level.

- **GrayLevelNonUniformity / Normalized** – heterogeneity of gray levels across zones.
- **GrayLevelVariance** – variance of gray levels.
- **HighGrayLevelZoneEmphasis** – zones with high gray levels.
- **LargeAreaEmphasis** – large zones (homogeneous areas).
- **LargeAreaHighGrayLevelEmphasis / LowGrayLevelEmphasis** – large homogeneous zones with high/low intensity.
- **LowGrayLevelZoneEmphasis** – emphasizes low intensity zones.
- **SizeZoneNonUniformity / Normalized** – heterogeneity of zone sizes.
- **SmallAreaEmphasis** – emphasizes fine texture (small zones).
- **SmallAreaHighGrayLevelEmphasis / LowGrayLevelEmphasis** – small zones weighted by intensity.
- **ZoneEntropy** – randomness of zone size/gray-level distribution.
- **ZonePercentage** – ratio of zones to possible voxels.
- **ZoneVariance** – variance of zone sizes.

7. Neighborhood Gray Tone Difference Matrix) features

These describe differences between a voxel and its neighborhood.

- **Busyness** – how rapidly gray levels change between voxels.
- **Coarseness** – spatial rate of intensity change (high = uniform, low = fine texture).
- **Complexity** – complexity of texture variation.
- **Contrast_A** – local contrast (different from GLCM contrast).
- **Strength** – overall perceptual strength of texture.
